# Polymorphisms of glutathione S-transferases (GST) and thymidylate synthase (TS) – novel predictors for response and survival in gastric cancer patients

**DOI:** 10.1038/sj.bjc.6602891

**Published:** 2005-11-29

**Authors:** E Goekkurt, S Hoehn, C Wolschke, C Wittmer, C Stueber, D K Hossfeld, J Stoehlmacher

**Affiliations:** 1 Department of Haematology and Oncology, University Hospital Hamburg Eppendorf, University of Hamburg, Germany; 2 Department of Internal Medicine I, University Hospital Carl Gustav Carus, University Dresden, Germany; 3 Department of Pathology, University Hospital Hamburg Eppendorf, University of Hamburg, Germany

**Keywords:** glutathione S-transferase P1, thymidylate synthase, pharmacogenetics, gastric cancer, 5-FU/cisplatin

## Abstract

To evaluate the predictive value of a panel of gene polymorphisms involved in metabolism of 5-FU and cisplatin on clinical outcome in advanced gastric cancer patients. A total of 52 patients were enrolled in this study. DNA was extracted from paraffin-embedded tumour specimen. Genotypes were determined using PCR-RFLP. Median survival time was 6.0 months (95% CI 3.9;8.1). Overall response rate was 26%. Patients possessing the glutathione S-transferase P1-105 Valine/Valine (GSTP1-105VV) genotype showed a response rate of 67% compared to 21% in patients harbouring at least one GSTP1-105 Isoleucine (GSTP1-105I) allele (*P*=0.038). GSTP1-105VV patients demonstrated a significant superior median survival time of 15.0 months (95% CI 7.8;22.0) compared to 6.0 months (95% CI 5.1;7.0) in patients with at least one GSTP1-105I allele (*P*=0.037). Patients possessing a favourable thymidylate synthase (TS) genotype (2R/2R, 2R/3RC, 3RC/3RC) experienced a superior survival time of 10.2 months (95% CI 5.1;15.3) compared to 6.0 months (95% CI 5.0;7.0) in patients with unfavourable TS genotypes (*P*=0.099). Patients harbouring the GSTP1-105II genotype and one of the unfavourable TS genotypes showed an inferior median survival time of 6.0 months (95% CI 3.9;8.1) compared to 11 months (95% CI 6,23;15,77) in patients with either GSTP1-105VV or a favourable TS genotype (*P*=0.044). Testing for TS and GSTP1 polymorphisms may allow identification of gastric cancer patients who will benefit from 5-FU/cisplatin chemotherapy, sparing others the side effects of this chemotherapy.

Worldwide, gastric cancer is the fourth common type of cancer and the second most frequent cause of death from cancer. The median survival time of patients with advanced gastric cancer ranges approximately from 7.5 to 12 months (Parkin, 2001). 5-FU/cisplatin has been referred to as a standard chemotherapy in gastric cancer patients for many years. During the last few years additional drugs were introduced into chemotherapy regimens for gastric cancer such as oxaliplatin, the taxanes and irinotecan. Results from phase II studies indicate a promising efficacy and managable toxicity profile for combination chemotherapies including these new substances (Al-Batran *et al*, 2004; Bouche *et al*, 2004; Chao *et al*, 2004; Lee *et al*, 2004; Park *et al*, 2004; Souglakos *et al*, 2004; Van Cutsem, 2004). One of the remaining challenges is the development of predictive marker profiles to identify patients who will derive both minimal toxicity and maximum benefit from certain chemotherapy.

There is a growing body of evidence suggesting that genetic polymorphisms in genes involved in metabolism, signalling, transport, DNA-repair and cellular response pathways all contribute to inter-patient variability of drug response and toxicity. Pharmacogenetic analyses appear to be a promising tool to develop individualised treatment plans.

Fluoropyrimidines remain an important drug in the chemotherapeutic treatment of gastric cancer. Decreased levels of the target enzyme thymidylate synthase (TS) have been repeatedly associated with superior clinical outcome in gastrointestinal cancers, including stomach cancer (Leichman *et al*, 1995; Boku *et al*, 1998; Shirota *et al*, 2001). TS catalyses the formation of thymidylate, a source of DNA replication, from dUMP (Danenberg, 1977). A repeat polymorphism within the 5′ untranslated region (UTR), that alters TS expression was correlated with response and survival in colorectal cancer patients receiving 5-FU in several studies (Horie *et al*, 1995; Kawakami *et al*, 1999; Iacopetta *et al*, 2001; Pullarkat *et al*, 2001; Marsh *et al*, 2001). Recently, a novel functional G/C single nucleotide polymorphism (SNP) within this repeat polymorphism was identified (Kawakami and Watanabe, 2003; Mandola *et al*, 2003). This G/C SNP causes a disruption of the binding site of the transcription factor USF-1 thereby altering TS expression. A recent report in colorectal cancer indicates the clinical significance of the G/C SNP (Marcuello *et al*, 2004). Finally, a 6-bp deletion polymorphism within the 3′ UTR of the TS gene has been described (Ulrich *et al*, 2000) that also alters TS expression, although the exact underlying mechanism remains unclear at this time (Mandola *et al*, 2004). In addition to polymorphisms of the TS gene, the common MTHFR-C677T polymorphism has been associated with response to 5-FU in colorectal tumours (Cohen *et al*, 2003; Etienne *et al*, 2004). Methylene tetrahydrofolate reductase (MTHFR) plays a central role in folate metabolism. The substrate for MTHFR, 5,10-MTHF, is used for the synthesis of dTMP by TS.

Glutathione-S-transferases are crucial for the cell defence system. These phase II detoxification enzymes are involved in detoxification of a variety of chemotherapeutics including platinum. The GSTP1 isoform has been detected at high levels within the gastrointestinal tract. In addition, *in vitro* analyses revealed a significant association between high GSTP1 expression of tumour cells and decreased sensitivity to platinum agents (Oguri *et al*, 2000). An A/G SNP located within the substrate-binding domain of GSTP1, at position +313 within exon 5, results in an amino-acid substitution of Isoleucine by Valine (Ile105Val). This polymorphism significantly influences enzyme activity (Zimniak *et al*, 1994) and is linked to clinical outcome of patients who received platinum-based chemotherapy (Stoehlmacher *et al*, 2002, 2004). GSTM1 and GSTT1 deletion polymorphisms (null genotypes) have also been reported to be associated with diminished GST enzyme activity (London *et al*, 2000).

Genes of the nucleotide-excision-repair (NER) pathway plays a key role in recognition and repair of damaged DNA caused by platinum compounds. Important members of the NER pathway, namely excision-repair-cross-complementing gene 1 and 2 (ERCC1 and ERCC2), the latest also named Xeroderma pigmentosum complementation group D (XPD) exhibit several polymorphic sites. Functional polymorphisms of both genes (ERCC1 – C118T and ERCC2 – Gln751Lys) have been demonstrated to impact clinical outcome of patients receiving platinum-based chemotherapy (Shen *et al*, 1998; Park *et al*, 2001; Isla *et al*, 2004).

In the current analysis, we studied a panel of nine genetic polymorphisms within seven genes (TS, MTHFR, GSTP1, GSTT1, GSTM1, ERCC1, ERCC2) involved in the metabolism of cisplatin and 5-FU as well as genes of the NER. We tested the hypothesis whether these polymorphisms, alone or in combination, may have the potential to predict response and survival in patients with advanced gastric cancer receiving 5-FU/cisplatin combination chemotherapy.

## PATIENTS AND METHODS

### Subjects

All patients included in this study had advanced gastric cancer and were treated between 2002 and 2004 at the Department of Oncology at the University Hospital Hamburg Eppendorf, Germany. All patients received at least one complete cycle of 5-FU/cisplatin/FA as first-line chemotherapy. One cycle consisted of biweekly cisplatin 50 mg m^−2^ (1 h infusion) and weekly FU 2 g m^−2^ (24 h continuous infusion) and FA 500 mg m^−2^ (2-h intravenous infusion) for 6 weeks. Response evaluation was performed every 6 weeks using CT scanning based on RECIST criteria. Responses were confirmed after 4 weeks. All patients agreed to perform genotype analyses in this study.

### Extraction of DNA

Paraffin-embedded tumour specimens for genotype analyses were available from 52 patients with advanced gastric cancer. Genomic DNA was extracted from representative sections (at least 80% of tumour infiltration) of formalin-fixed, paraffin-embedded tumour specimen using the QiaAmp kit (Qiagen, Valencia, CA, USA). Representative tumour sections were selected after evaluation (light microscopy) by the pathologist (CW).

### Study design

Genotyping was carried out by using PCR-based RFLP protocols as described previously. The following genetic polymorphisms were analysed: glutathione S-transferase (GSTP1 – Ile105Val, GSTT1 and GSTM1 deletion), thymidylate synthase (TS-5′ UTR 2R/3R; TS-5′ G/C; TS-3′ UTR 1494del6), methylene-tetrahydofolate-reductase (MTHFR – C677T), excision repair cross-complementing gene 1 and 2 (ERCC1 – C118T and ERCC2 – Gln751Lys) (Frosst *et al*, 1995; Arand *et al*, 1996; Lunn *et al*, 2000; Mandola *et al*, 2003; Stoehlmacher *et al*, 2004). The PCR reaction volume was 25 μl and restricted PCR fragments were separated on a 3% agarose gel. PCR/RFLP analyses were performed by a technician blinded for the clinical data. For quality purposes 20% random samples of each genotype were repeated.

A comprehensive chart review was performed to obtain all necessary demographic and clinical information including age, sex, involved metastatic sites, objective tumour response rate and survival. Survival was measured from the beginning of 5-FU/cisplatin chemotherapy until death from any cause.

### Statistical analyses

Statistical analyses were carried out using SPSS for Windows (Version 13.0). Fisher's exact test (two-sided) and *χ*^2^ test were used to assess the association between each genotype and clinical response to 5-FU/cisplatin chemotherapy. The log-rank test (Miller, 1981) and Kaplan–Meier plots (Kaplan and Meier, 1958) were used to evaluate the association of genotypes and overall survival. Statistical significance was interpreted as *P*<0.05.

## RESULTS

In this retrospective study, 52 Caucasian patients consisting of 18 females (35%) and 34 males (65%) were analysed. Patients’ demographics and disease characteristics at baseline are given in Table 1. The median follow-up time was 6.2 months (95% CI 4.6; 7.4). Median survival time was 6.0 months (95% CI 3.9; 8.1). None of these patients were referred for definitive surgery after application of chemotherapy. Two patients were excluded from response evaluation, because the response confirmation after 4 weeks was not documented in the medical chart. In all, 26% of patients (13/50) showed a response (partial response (PR) or complete response (CR)) to 5-FU/cisplatin chemotherapy. The majority of patients (37/50, 74%) demonstrated stable disease (SD) or progressive disease (PD).

Genotypes could be determined as follows: GSTP1 50/52 (96%), GSTM1 and GSTT1 52/52 (100%), ERCC1 51/52 (98%), ERCC2 51/52 (98%), MTHFR-677 52/52 (100%), TS-5′ UTR 45/52 (87%), TS-5′ UTR G-C SNP 43/52 (83%) and TS-3′ 41/52 (79%). Distribution of genotypes and responses of the available 50 patients is given in Table 2.

### Association between GSTP1-105 polymorphism and response and survival

GSTP1-105 genotype analyses were successful for 50 patients of which 48 were eligible for response analyses.

For association analysis of genotype and response to chemotherapy patients with CR and PR were determined ‘responders’ and patients with SD and PD were referred to as ‘nonresponders’. According to previous data (Stoehlmacher *et al*, 2004) patients were divided into a favourable (homozygous GSTP1-105Val) and an unfavourable genotype group (heterozygots and homozygous GSTP1-105Ile). Patients possessing the GSTP1-105 Val/Val genotype showed a significant superior response rate of 67% (4/6) compared to only 21% (9/42) in patients harbouring at least one GSTP1-105Ile allele (*P*=0.038, Fisher's exact test) (Table 3). None of the patients homozygous for the GSTP1-105Val allele showed disease progression.

**Figure 1:**
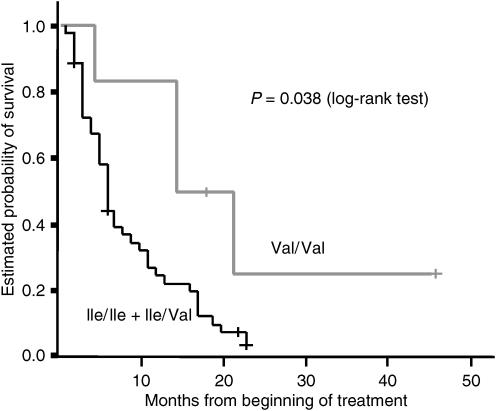
Association between GSTP1-105 genotypes and overall survival in patients with advanced gastric cancer.

Patients with GSTP1-105 Val/Val genotype demonstrated significant superior median survival time with 15.0 months (95% CI 7.8; 22.0) compared to only 6.0 months (95% CI 5.1; 7.0) for patients with at least one GSTP1-105Ile allele (*P*=0.038, log-rank test) (Figure 1).

### Association between TS polymorphisms and outcome to 5-FU/cisplatin chemotherapy

Both, the TS-3′ UTR (6-bp deletion) polymorphism and the polymorphisms in the 5′ UTR (28bp repeat + G/C SNP) were not associated with response to chemotherapy.

However, combined analysis of polymorphisms in the TS-5′ UTR showed a trend for superior survival in patients harbouring a favourable TS-5′ UTR genotype. The favourable TS-5′ UTR genotype group included those genotypes that have been linked to decreased TS expression; 2R/2R, 2R/3RC, 3RC/3RC. Whereas the unfavourable group consisted of TS genotypes associated with elevated TS levels: 2R/3RG, 3RC/3RG and 3RG/3RG (Mandola *et al*, 2003; Marcuello *et al*, 2004). The favourable group experienced a superior median survival time of 10.2 months (95% CI 5.1; 15.3) compared to only 6.0 months (95% CI 5.0; 7.0) in the unfavourable genotype group (*P*=0.099, log-rank test).

### Combined analysis of GSTP1 and TS-5′ genotypes and survival

Based on the results of superior survival observed for the favourable GSTP1-105 genotype group and the favourable TS-5′ UTR genotype group in the univariate analysis, a combined analysis for survival was performed.

**Figure 2:**
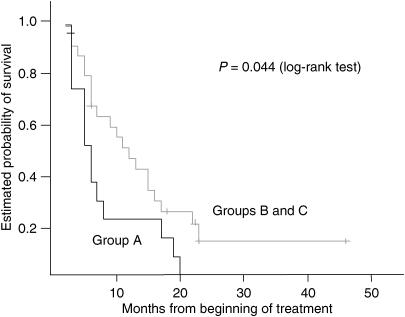
Association between combined analyses of GSTP1-105 and TS-5′ UTR polymorphisms including the TS-5′ G-C SNP and overall survival among patients with advanced gastric cancer. Group A includes patients with only unfavourable genotypes in both genes (*n*=15). Group B includes either patients homozygous for the GSTP1-105 Val variant or patient with a favourable TS-5′ UTR genotype (*n*=23) and group C patients are homozygous for GSTP1-105 Val and possess a favourable TS-5′ UTR polymorphism (*n*=3).

All genotypes (GSTP1-105, TS-5′ repeat and TS5′-SNP) could be identified in 41 of 52 patients (79%). Patients were divided into three groups: patients possessing neither GSTP1 Val/Val nor one of the favourable TS-5′ UTR genotypes (group A, *n*=15 (37%)), patients harbouring either GSTP1 Val/Val or a favourable TS-5′ UTR genotype (group B, *n*=23 (56%)) and patients with GSTP1-105 Val/Val and a favourable TS-5′ UTR genotype (group C, *n*=3 (7%)). Owing to the small size of group C, this group was combined with group B for survival analyses. Group B and C patients combined demonstrated a superior median survival time of 11.0 months (95% CI 6,23; 15,77) compared to only 6.0 months (95% CI 3.9; 8.1) for group A patients (*P*=0.044, log-rank test, Figure 2). Two patients of group C were still alive at time of analyses with a survival time of 18 and 22 months, respectively. The third patient of this group died 15 months after start of 5-FU/cisplatin chemotherapy.

No associations between any of the polymorphisms TS-3′ 1494del6, MTHFR – C677T, GSTM1, GSTT1, ERCC1 – C118T or ERCC2 – Gln751Lys and response (Table 2) or overall survival were found.

## DISCUSSION

We attempted to identify predictive genetic polymorphisms for response and survival to 5-FU/cisplatin chemotherapy in patients with advanced gastric cancer. In our study, the GSTP1-105 polymorphism was identified as a predictive and a prognostic marker in gastric cancer patients. Cisplatin (CDDP), widely used in the treatment of solid tumours, is in part detoxified by glutathione S-transferase P1 (GSTP1). The GSTP1-105Val allele was significantly associated with increased response rate and superior survival in the current study. The insufficient formation of CDDP-Glutathione (CDDP-GSH) adducts, the detoxified form of CDDP, was postulated as mechanism for the protective effect of the GSTP1-105Val variant. Goto *et al* (1999) provided evidence for the direct involvement of GSTP1 in the detoxification of CDDP by forming CDDP-GSH adducts. Transfection of GSTP1 cDNA into human colon cancer cells resulted in an increase of GSTP1 expression and an augmentation of DDP-GSH adduct formation (Goto *et al*, 1999). In addition, transfection analyses reported by Ban *et al* (1996) showed an association between GSTP1 and both acquired and intrinsic resistance to cisplatin (Ban *et al*, 1996). As a result of impairment of the GSTP1 substrate binding capacity caused by the underlying A → G substitution, patients homozygous for the GSTP1-105 valine variant may accumulate more drug compared to patients possessing one or two wild-type alleles (Watson *et al*, 1998). These results are in agreement with reports linking high GSTP1 mRNA expression to improved survival in oesophageal cancer patients. Among patients with oesophageal cancer, who received a 5-FU/cisplatin combination chemotherapy and radiation, those patients with decreased intratumoral GSTP1 levels demonstrated significant superior survival (Joshi *et al*, 2005). Additionally, reports in colorectal cancer patients demonstrated a significantly improved time to progression and overall survival for carriers of the GSTP1-105Val allele after 5-FU/oxaliplatin chemotherapy (Stoehlmacher *et al*, 2002, 2004). In contrast, McLeod *et al* (2003) did not observe a correlation between the GSTP1-Ile105Val polymorphism and response to oxaliplatin-based chemotherapy in colorectal cancer patients (McLeod *et al*, 2003). These controversial results in colorectal cancer may indicate a lower impact of the GSTP1-Ile105Val polymorphism in oxaliplatin-based chemotherapy regimens. Cabelguenne *et al* (2001) could not observe a correlation between GSTP1 genotypes and response to cisplatin neoadjuvant chemotherapy in head and neck squamous cell cancer (HNSCC) patients. However, they described that low plasmatic GSTP1 levels conferred to a 2.3-fold increased relative risk of complete response in HNSCC patients (Cabelguenne *et al*, 2001).

In summary, the results we report here are in strong agreement with the current understanding of GSTP1 involvement in cisplatin detoxification. Our findings support the hypothesis that increased cisplatin sensitivity may in part be due to impaired GSTP1 enzyme function. However, a critical review of our results in conjunction with the current literature revealed that analysis of only the GSTP1 polymorphism does not represent a valid approach to distinguish responders from nonresponders to a combination chemotherapy of 5-FU and cisplatin. Reasons are the static character that is inherent to a polymorphism and the complexity of metabolising steps for chemotherapeutics. Therefore, we investigated a panel of nine genes involved in 5-FU and cisplatin metabolism.

Considering the known interactions between GST genotypes, we also analysed GSTM1 and GSTT1 genotypes, but did not find any correlation with clinical characteristics. Polymorphisms in ERCC1 and ERCC2 also did not show any correlation with clinical outcome in this study. One reason for this result maybe the limited number of patients in the retrospective analysis.

For a more accurate prediction of response and survival to the combination treatment of 5-FU and cisplatin, we also aimed to explore possible associations between polymorphisms in fluoropyrimidine metabolism and clinical outcome.

The active metabolites of fluoropyrimidines exert their antitumour activity mainly through TS inhibition. This reaction is facilitated by the formation of a ternary complex consisting of TS, 5,10-Methylene THF and 5-fluoro-dUMP (Peters *et al*, 2002). There is growing evidence for functionally important polymorphic variations within the TS gene. The 28-bp repeat and the G/C SNP within the 5′ UTR of the gene as well as the 6bp deletion polymorphism in the 3′ UTR, all have been described to alter expression of TS (Pullarkat *et al*, 2001; Kawakami and Watanabe, 2003; Mandola *et al*, 2003, 2004). In the current study, patients who possessed a TS 5′ genotype associated with low TS mRNA expression levels (Mandola *et al*, 2003) demonstrated a trend for superior survival time compared with patients harbouring TS genotypes known to be associated with high TS mRNA expression. This observation is in agreement with recent findings by Marcuello *et al* (2004) who revealed a significant association between clinical outcome to 5-FU-based chemotherapy in colorectal cancer and TS polymorphisms only if both TS polymorphisms within the 5′ UTR were analysed in conjunction (Marcuello *et al*, 2004). A trend between TS polymorphism and clinical outcome was observed by Ishida *et al* (2002) in gastric cancer patients who received oral fluoropyrimidine therapy. However, the authors did not include the novel G/C SNP that functionally transfers a TS 3R genotype into a TS 2R genotype in their analysis. Although the observed association between TS polymorphisms and outcome in our study did not reach significance, the results support the hypothesis that a comprehensive analysis of these TS polymorphisms is needed if associations between TS polymorphisms and response to fluoropyrimidine chemotherapy is explored. The reason that the observed effect did not reach statistical significance may be again due to the small number of patients.

Based on these findings, favourable and unfavourable genotype groups were defined to describe the clinical outcome to 5-FU/cisplatin combination chemotherapy in gastric cancer. We could demonstrate that a patient's benefit of 5-FU/cisplatin chemotherapy significantly increases with the number of favourable genotypes. A combined analysis may more accurately identify patients with maximum benefit from therapy. Although the number of patients possessing two favourable genotypes was small in our study their median survival time reached almost 24 months. These findings, if confirmed, may have a significant impact on the management of gastric cancer patients, since alternative drug combinations are becoming available.

Our study is limited by both its retrospective nature and the relatively small sample size. In addition, meanwhile more polymorphic genes involved in platinum and 5-FU metabolism have been identified (e.g. ERCC1-C8092A, MTHFR-A1298C), that may also impact the effect of these drugs. To confirm our findings from this pilot study and to improve the predictive value of pharmacogenetic analyses in gastric cancer patients, we are currently analysing a panel of 26 polymorphisms in genes of metabolising and DNA repair enzymes in a large, randomised, prospective phase III trial in advanced gastric cancer, involving more than 220 patients.

To conclude, this pilot study demonstrated that combined genotype analysis of GSTP1, TS-5′ 28bp and TS-5′ G → C polymorphisms may contribute to the selection of gastric cancer patients who would benefit most from 5-FU/cisplatin combination chemotherapy. To our knowledge, this is the first report demonstrating a predictive value of GSTP1 genotypes in regard to 5-FU/cisplatin combination chemotherapy in gastric cancer patients.

## Figures and Tables

**Table 1:** Patient's demographics and disease characteristics at baseline

	***n***	**%**
*Age (years)*	56	—
Range	27–82	—
		
*Gender*		
Male	34	65
Female	18	35
		
*Karnofski index*		
100–90	27	52
80–70	25	48
		
*Number of involved sites*		
1	7	13
2	18	35
⩾3	27	52
		
*Previous surgery*		
Yes	37	71
No	15	29
		
*Prior adjuvant chemotherapy*		
Yes	3	6
No	49	94

**Table 2:** Distribution of genotypes and responses

		**Responder**	**Nonresponder**	
	**Total n (%)**	***n* (%)**	***n* (%)**	***P*-value^a^**
All	50 (100)	13 (26)	37 (74)	—
*TS-5′UTR*				
2R/2R	13 (26)	2 (15)	11 (85)	
2R/3R	19 (38)	7 (37)	12 (63)	
3R/3R	12 (24)	3 (25)	9 (75)	0.400
Missing	6 (12)			
				
*TS-5′ UTR+G/C SNP*				
2/2, 2/3C, 3C/3C	23 (46)	5 (22)	18 (78)	
2/3G, 3C/3G, 3G/3G	20 (40)	7 (35)	13 (65)	0.334
Missing	7 (14)			
				
*TS-3′ 1494del6*				
−6/−6	12 (24)	5 (42)	7 (58)	
−6/6	23 (46)	6 (26)	17 (74)	
6/6	5 (10)	1 (20)	4 (80)	0.553
Missing	10 (20)			
				
*GSTP1–105*				
Ile/Ile	30 (60)	7 (23)	23 (77)	
Ile/Val	12 (24)	2 (17)	10 (83)	
Val/Val	6 (12)	4 (67)	2 (33)	0.060
Missing	2 (4)			
				
*GSTM1*				
GSTM1−	32 (64)	9 (28)	23 (72)	
GSTM1+	18 (36)	4 (22)	14 (78)	0.648
Missing	0 (0)			
				
*GSTT1*				
GSTT1−	38 (76)	8 (21)	30 (79)	
GSTT1+	12 (24)	5 (42)	7 (58)	0.156
Missing	0 (0)			
				
*ERCC1–118*				
C/C	5 (10)	2 (40)	3 (60)	
C/T	26 (52)	6 (23)	20 (77)	
T/T	18 (36)	5 (28)	13 (72)	0.727
Missing	1 (2)			
				
*ERCC2–751*				
Gln/Gln	12 (24)	3 (25)	9 (75)	
Gln/Lys	25 (50)	8 (32)	17 (68)	
Lys/Lys	12 (24)	2 (17)	10 (83)	0.607
Missing	1 (2)			
				
*MTHFR-677*				
C/C	28 (56)	10 (36)	18 (64)	
C/T	20 (40)	2 (10)	18 (90)	
T/T	2 (4)	1 (50)	1 (50)	0.099
Missing	0 (0)			

50 of 52 patients (96%) were evaluable for response. Two pts were excluded for response evaluation due to missing response confirmation (MRC). For association analysis of genotype and response to chemotherapy patients with complete response (CR) and partial response (PR) were determined ‘responder’ and patients with stable disease (SD) and progressive disease (PD) were referred to as ‘non-responder’.

a *χ*^2^ test.

**Table 3:** Association between GSTP1-105 genotype and response to 5-FU/cisplatin chemotherapy in patients with advanced gastric cancer

		**Responders**		**Nonresponders**	
	**Total (*n*)**	**No.**	**%**	**No.**	**%**
Genotype					
Val/Val	6	4	67	2	33
Val/Ile +					
Ile/Ile	42	9	21	33	79

*P*=0.038 (Fisher's exact test, two-sided) for genotype – response association.
